# The socio-economic status gradient in median lifespan by birth cohorts: Evidence from Dutch Olympic athletes born between 1852 and 1947

**DOI:** 10.1371/journal.pone.0226269

**Published:** 2019-12-11

**Authors:** Adriaan Kalwij

**Affiliations:** Utrecht University, School of Economics, Utrecht, The Netherlands; University of Malaya, MALAYSIA

## Abstract

This paper quantifies the socio-economic status (SES) gradient in median lifespan for three birth cohort groups. For this, mortality models were estimated using unique data on the SES of Dutch Olympic athletes born between 1852 and 1947, and who were followed until their death (or December 2018). The empirical findings show that for the older birth cohorts (1852–1899) there were no significant differences in median lifespan between SES groups. For the middle cohorts (1900–1919), the low SES athletes had a significantly lower median lifespan of five years less compared to medium SES athletes and the median lifespans of high and medium SES athletes did not differ significantly. For the younger cohorts (1920–1947), large and statistically significant differences were found between the three SES groups: low SES athletes had a median lifespan of about six years lower than medium SES athletes, while high SES athletes had an almost five years higher median lifespan compared to medium SES athletes. These new findings, which can be reconciled with the existing literature, suggest a strong steepening of the population SES-lifespan gradient over time in the Netherlands.

## Introduction

Over the past few decades a steepening of the contemporaneous socio-economic status (SES) gradient in the mortality rate has been documented for Europe and the United States. This is at the forefront in the ongoing policy debate on public health [1–4]. While the empirical evidence on the steepening of the SES-mortality gradient is often expressed in terms of period life expectancies, it does not allow for an analysis of the SES-lifespan gradient as that would require following individuals, for whom their SES is known, throughout their lives. This paper takes the latter approach and, therefore, adds to the literature by providing empirical evidence on the development of the SES-lifespan gradient for individuals born in the second half of the 19^th^ and first half of the 20^th^ century. The empirical evidence could provide further insight into whether lifespan inequalities associated with socio-economic status can be related to how society is organized, for instance through social policy [3]. In the Netherlands, which is the country this paper investigates, the welfare state that was strengthened during the 20^th^ century (e.g. in introducing disability and long-term care insurances, universal health care, and state pensions) could, arguably, have weakened the SES-lifespan gradient over time.

A few studies have considered the (contemporaneous) SES-mortality gradient over a long period of time and have found that it can vary in strength. The most comprehensive study in terms of covering a long period with detailed data was carried out for Sweden. It showed that the SES-mortality gradient was absent in the first half of the 20^th^ century and then emerged in the second half [5]. For England and Wales, social class inequality in mortality declined in the 1920s, was still present in the period 1930–1932, and increased again during the 1950s and 1960s. By 1970, this inequality was more pronounced than at the start of the 20^th^ century [6,7]. For the U.S. there have also been periods of substantially less steep SES-health gradients than we find nowadays, and socio-economic differences decreased in the first half of the 20^th^ century [3,8]. For the Netherlands, mortality was relatively high among lower social classes around 1900; by around 1950 this mortality gradient was no longer present, while around 1960 it resurfaced [9]. This long-term pattern is in line with the findings of two other Dutch studies, that during the period 1820–1920 there were mortality differences between SES groups, but that they decreased over time [10], just to see an SES-mortality gradient increase again between the 1950s and 1980s [11]. As far as I know, the existing literature has not examined how these changes in the contemporaneous SES-mortality gradient have affected generations’ SES-lifespan gradients.

This paper, therefore, quantifies the SES-lifespan gradients for three birth cohort groups. For this, mortality models have been estimated using unique data on the SES of Dutch Olympic athletes born between 1852 and 1947, and who have been followed until their death (or December 2018). The data contains one measurement of athletes’ SES based on their occupations.

The main empirical findings suggest that there has been a strong steepening of the SES-lifespan gradient over time. There are no significant differences in median lifespan between SES groups for the older birth cohorts (1852–1899). For the middle cohorts (1900–1919), low SES athletes had a significant five years lower median lifespan than medium SES athletes and median lifespans of high and medium SES athletes did not differ significantly. For the younger cohorts (1920–1947), low SES athletes had about a six years lower median lifespan, while high SES athletes had an almost five years higher median lifespan than medium SES athletes.

## The data

For Dutch Olympic athletes who participated in the 1900 to 2012 Olympic Games we have data on their gender, particular kind of sport, date of birth, and if deceased before 2012, date of death [12]. This data has been supplemented with the dates of death of athletes who died between 2012 and 2018 [13]. Age and gender specific annual population death rates have been retrieved from the Human Mortality Database and the Royal Dutch Actuarial Association [14,15]. These death rates have been used to compare the lifespan distribution of Olympic athletes with the population lifespan distribution (S1 Fig). Further, information is available on athletes’ occupations before or, most often, after they participated in the Olympics [12]. This measurement of athletes’ SES is taken as a proxy for their lifetime SES.

The Netherlands did not participate in the 1896 Olympics, and most athletes who participated after the 1964 Olympics are still alive. Our data, therefore, is restricted to athletes that participated in the 1900 to1964 Olympic Games. The birth or death dates of 13 of the 1163 athletes are unknown, or the available records could not confirm that they were still alive in December 2018, and the SES of a further 216 athletes (mostly women) is unknown. These athletes have been dropped from the sample. Conditional on gender, unknown SES is not significantly related to lifespan. The final sample thus consists of 934 athletes. These athletes were followed from the age they participated in the Olympics, which on average is 26, until their death or December 2018 (whichever came first). About 85% of the athletes were deceased by December 2018. Further, the sample contains only 57 female athletes, which prevents a thorough analysis by gender (S1 Table reports these statistics according to particular Olympic Games).

In Table 1, the top two rows give a comparison of Dutch Olympic athletes’ lifespan distribution and that of the general population. This reveals that the median lifespan of athletes is about two years more than that of the general population (S1 Fig shows this comparison by Olympic Games). The gender difference in median lifespan among athletes is nine years in favor of women and also, not reported here, higher than in the general population. Table 1 further shows that the lifespan distribution shifts upwards with an increase in the year of the Olympics. S2 Table reports on the lifespan distribution by type of sport and by medal position (gold, silver, or bronze).

**Table 1:** Lifespan distributions for the general population and for Olympic athletes by gender, Olympic Games in which they first participated, and socio-economic status.

	Number of athletes	Lifespan (in years)		
		25th percentile	50th percentile	75th percentile
General population^a^		66	76	84
All athletes	934	67	78	86
Male athletes	877	66	78	86
Female athletes	57	78	87	91
*Year(s) of the Olympic Games*^b^				
1900/06/08	144	63	70	82
1912/20	113	65	74	82
1924	111	64	75	82
1928	132	66	77	84
1932/36	128	70	80	88
1948	85	69	81	88
1952/56	57	75	84	-
1960	77	76	-	-
1964	87	-	-	-
*Socio-economic status (SES)*				
Low SES	153	65	76	84
Medium SES	398	70	80	87
High SES	383	66	78	87

Log-rank tests reject equality of survivor functions by gender (p-value = 0.000), by Olympic Games (p-value = 0.000), and by SES (p-value = 0.016).

^a^ Based on a weighted average of annual death rates of the athletes’ national cohorts and gender, conditional on reaching 26 (the average age at which athletes participated in the Olympics).

^b^ Not all athletes that participated in the more recent Olympics had died by December 2018 (S1 Table). Therefore, not all quartiles of the lifespan distribution can be reported by Olympic Games.

The information on athletes’ SES has been summarized in fourteen categories based on occupation, and sometimes professional higher education for becoming medical doctors, engineers, and lawyers. The numbers of observations for each of these fourteen categories are too small for a thorough analysis and have, therefore, been aggregated into three categories representing low, medium, and high SES. Details on the classification are given in S2 Table, as are the lifespan distributions for each of the fourteen categories. Table 1 shows that high and medium SES athletes have a longer median lifespan than low SES athletes. It also shows that the median lifespan of high SES athletes is two years lower than that of medium SES athletes. Explanations for such differences could be related to, for instance, the athlete’s type of sport and birth cohort, which relates to the year in which an athlete participated in the Olympics. Our empirical analysis aims to control for such factors.

## Mortality model

The relationship between SES and lifespan, after controlling for other observed characteristics, is estimated using a proportional hazard rate model [16]. Because lifespan is measured in full years, I estimated a discrete-time proportional hazard rate model and used annual population death rates to flexibly control for gender differences in mortality, an age gradient, time effects, and different gender, age, and time effects across cohorts [17]. To be more specific, the annual mortality rate of Olympic athlete *i* at age *a* with covariates **x**_*i*_(*a*) is defined as follows:

$$\lambda^{d}\left(a|c_{i},g_{i},x_{i}\left(a\right),\beta \right)=1-\text{exp}\left(-\mu_{c_{i}g_{i}}\left(a\right)\text{exp}\left(x_{i}{\left(a\right)}^{\prime }\beta \right)\right)$$ (1)

where $\mu_{c_{i}g_{i}}\left(a\right)$ is the annual death rate among the general population with the same age *a*, birth year *c*_*i*_ and gender *g*_*i*_ as athlete *i*. The vector **x**_*i*_(*a*) includes a constant, and the covariates are allowed to vary with age to accommodate athletes who competed at more than one Olympics. Based on the underlying continuous-time hazard rate, a coefficient corresponding to a dummy variable (*β*_*k*_) is interpreted as a 100x(exp(*β*_*k*_)-1) percentage increase in the mortality rate for a unit increase in the dummy variable (from 0 to 1). For small values of *β*_*k*_, the coefficient times 100 is a good approximation of the percentage effect. All covariates except age are dummy variables.

Gender and age are included as covariates to allow for variation between Olympic athletes and the general population in gender-based mortality differences and the mortality-age gradient. The model includes a covariate for whether the athlete participated in more than one Olympics, to control for possible adverse health effects due to a longer period of training [18]. Further, because the selection of Olympic athletes is likely to vary over time because of many sport types being professionalized by the Dutch Olympic Committee [19], controls for the different Olympics are included. Finally, I controlled for different sports and which medal position was achieved, as these have been shown to be related to mortality risk, and might additionally be related to SES [17,20–22].

(Eq 1) is estimated by Maximum Likelihood for three birth cohort groups: the older (1852–1899), middle (1900–1919) and younger birth cohorts (1920–1947). The division into these cohort groups was made in such a way that there are about equal numbers of athletes in each group. In doing so, I took into account the findings of previous studies that the contemporaneous SES-mortality gradient had decreased around the turn of the 19th century, and has increased since around the middle of the 20th century. Therefore, 1900 was chosen as an upper bound for the older cohort group to distinguish between athletes born in the 19th and in the 20th century, and 1920 was chosen as a lower bound for the younger cohort group. The latter group members have spent most of their adult or working lives following their participation in the Olympics, in the second half of the 20th century.

Next, the SES-lifespan gradient is predicted for each of these groups based on the estimated SES-mortality gradient by birth cohort group. That changes in the SES-lifespan gradient can be driven by both cohort and period effects, is acknowledged. Besides medical innovations that continuously improved individuals’ health, they could also have experienced events which affected their health in the short and long term. For instance, the Dutch famine of 1944–45 had short and long term health consequences [23]; increases in compulsory schooling years in the Netherlands during the 20^th^ century affected lifespans [24]; the 1964 US Surgeon General’s report which confirmed that smoking caused lung cancer [25] may have brought individuals to stop smoking, which came with health benefits; and, business cycles have had both short and long term effects on health [26,27]. Likewise, the introduction of universal health care is likely to have positively affected individuals’ lifespans [28]. As the extent to which individuals’ health was affected by such events could depend on their SES—see, for instance, the case of smoking behavior across cohorts and SES [29]—there could have been long-lasting effects on the SES-mortality gradient throughout individuals’ lives. These effects have been modelled as cohort effects in the SES-mortality gradient, i.e. (Eq 1) is estimated by birth cohort group.

## Results

The estimation results of (Eq 1) for the three birth cohort groups (the older, middle and younger birth cohorts) summarized in Table 2 suggest a steepening of the SES-mortality gradient with an increase in the year of birth: There is no significant SES-mortality gradient for the older cohorts, a marginally significant gradient for the middle cohorts, and a significant and strong negative gradient for the younger cohorts. These differences in the SES-mortality gradient between cohort groups are statistically significant at the 5% level (S3 Table). For athletes born between 1920 and 1947 (the younger cohorts), the estimated SES-mortality gradient translates into a 38% lower mortality rate (100x(exp(-0.48)-1)) for high SES athletes and a 76% higher mortality rate for low SES athletes, compared to the mortality rate of medium SES athletes.

**Table 2:** Estimation results of (Eq 1) by birth cohort groups.

	Older birth cohorts 1852–1899	Middle birth cohorts 1900–1919	Younger birth cohorts 1920–1947
	Coeff.	Coeff.	Coeff.
Covariate: Socio-economic status (SES) Reference category: Medium SES	(SE) [p-value]	(SE) [p-value]	(SE) [p-value]
Low SES	-0.21	0.44**	0.57**
	(0.17) [0.22]	(0.18) [0.02]	(0.25) [0.03]
High SES	0.12	0.06	-0.48**
	(0.14) [0.39]	(0.14) [0.68]	(0.23) [0.04]
Number of medalists	352	305	277
Number of parameters	19	19	18
Value log-likelihood function	-1395.28	-1219.05	-634.64
Null hypothesis; cells contain p-values			
No gender and age associations	0.18	0.56	0.87
No associations with the different Olympics	0.35	0.11	0.88
No associations with SES	0.23	0.05**	0.00***
No association with participation in multiple Olympics	0.08	0.38	0.14
No associations with medal position	0.78	0.27	0.23
No associations with types of sport	0.11	1.00	0.23
Proportional hazard specification of SES associations	0.23	0.09*	0.80

All mortality rate models control for annual population death rates (by age, year, and gender), SES, gender, age, Olympics, participation in multiple Olympics, medal position, and type of sport. Top half of the table: The coefficient estimates (Coeff.) are of parameter vector β from (Eq 1) and standard errors are in parentheses (SE). The reported p-values additionally provide insight into possible issues related to multiple comparisons tests being carried out. Levels of significance:

*** p<0.01

** p<0.05

* p<0.1.

The p-values in the bottom half of the table suggest no significant associations of the covariates other than SES with the mortality rate. Further, and in favor of the proportional hazard assumption imbedded in (Eq 1), interactions between age and SES are jointly insignificantly associated with the mortality rate at the 5% level for each of the three birth cohort groups (Table 2; last row).

The median lifespan predictions of Table 3 are based on the estimates of (Eq 1). Median and not average lifespans are reported as some athletes in the most recent cohorts are still alive (Table 1). Table 3 (top panel) shows, in line with the well-documented increase in population longevity over time, that median lifespan in the general population increased from 75 years for the older cohorts (1852–1899) to 80 years for the younger cohorts (1920–1947). This increase is higher among medium SES athletes, at about 73 years for the older cohorts through to 85 years for the younger cohorts. Consequently, the difference in median lifespan between medium SES athletes and the general population increased from an insignificant difference for the older cohorts to about a five years marginally significant difference for the younger cohorts (Table 3; middle panel). One possible explanation for this faster increase in median lifespan for Olympic athletes compared to the general population is that, from the perspective of performance and, arguably, also health, Olympic athletes became a more select group over time due to the professionalization of sport by the Dutch Olympic Committee [19].

**Table 3:** Predicted median lifespans in the population by SES and birth cohort groups.

	Older birth cohorts 1852–1899	Middle birth cohorts 1900–1919	Younger birth cohorts 1920–1947
	Prediction	Prediction	Prediction
Median lifespan of the general population (in years)	75.00	76.00	80.00
Median lifespan of medium SES athletes (in years)	72.88	78.03	84.58
	Prediction	Prediction	Prediction
*Differences between SES groups (in years)*	(SE)	(SE)	(SE)
	[p-value]	[p-value]	[p-value]
Median lifespan of medium SES athletes	-2.12	2.03	4.58*
minus that of the general population	(1.97)	(2.66)	(3.33)
	[0.281]	[0.445]	[0.169]
Median lifespan of low SES athletes	2.65	-5.18**	-6.03**
minus that of medium SES athletes	(2.03)	(2.14)	(2.96)
	[0.191]	[0.015]	[0.041]
Median lifespan of high SES athletes	-1.37	-0.97	4.69*
minus that of medium SES athletes	(1.64)	(1.64)	(2.48)
	[0.402]	[0.554]	[0.058]

Median lifespan predictions are based on the estimation results of (Eq 1). Point predictions are provided, and for the differences in lifespans between groups standard errors are given in parentheses and p-values in brackets. Median lifespan predictions are conditional on having reached age 26 (i.e. the average age at which athletes participated in the Olympics). Levels of significance:

*** p<0.01

** p<0.05

* p<0.1.

The differences in median lifespans between low and medium SES athletes, and between high and medium SES athletes, are not significant in the older cohorts (Table 3; bottom panel, first column). For the middle cohorts there is a significant five years lower median lifespan for low SES athletes compared to medium SES athletes, and no significant difference in the median lifespans of high and medium SES athletes. For the younger cohorts there are large and statistically significant differences between the three SES groups, namely about a six years lower median lifespan for low SES athletes, and an almost five years higher median lifespan for high SES athletes compared to medium SES athletes. S4 Table provides results of statistical tests on the differences in the various relative median lifespans between cohort groups.

## Discussion

This paper has presented new empirical evidence in favor of the population SES-lifespan gradient steepening with an increase in the year of birth in the Netherlands. While similar evidence is not available from other studies, this finding can be reconciled with the empirical evidence on the contemporaneous SES-mortality gradient from previous Dutch studies of a decreasing gradient around the turn of the 19^th^ century and an increase in the second half of the 20^th^ century [9–11]. In addition, the difference in median lifespan of almost eleven years between low and high SES athletes for the younger cohorts (Table 3) can be reconciled with the recent Dutch evidence of a nine-year difference in period life expectancy between low and high-income individuals [30].

### Data limitations

An important advantage of using data from Olympic athletes is that such data makes it possible to analyze the SES-lifespan gradient for different birth cohorts, as it covers a very long period of time and most athletes are followed from early adulthood. Yet, as always, there are also drawbacks to using this particular sample. First, one can argue that this is a rather selective sample of individuals in relatively good health as they are elite athletes. If so, and if their excellent (innate) health made them “immune” to a SES-lifespan gradient, it would cause an underestimation of the population SES-lifespan gradient. Also, with a similar argument, the empirical finding that suggests relatively healthier athletes participated in the more recent Olympics (S1 Fig) could have led to an underestimation of the steepening of the SES-lifespan gradient.

Second, the SES of Olympic athletes might not reflect their true SES as they may have pursued a professional sport career as means of support. However, almost all Olympic athletes in our sample did not do so. They held regular jobs after having retired from the sport they represented in the Olympics [12]. The few exceptions are mainly cyclists for whom their sport provided a means of support for a limited number of years; after retiring from their cycling careers they then took up regular jobs. Excluding cyclists from the sample did not affect the main findings. Of course, having been an Olympic athlete *per se* might have boosted SES, but that would hold irrespective of SES based on occupation.

Third, the older, middle and younger birth cohort groups were formed based on relatively random criteria; e.g., choosing 1920 as the cut-off point between the middle and younger cohorts was not done on essential grounds. The number of athletes in the sample prevents a thorough analysis using more refined cohort groups to determine more precisely when the SES-mortality gradient emerged.

Fourth, although the main conclusion of a steepening of the SES-lifespan gradient over time is based on statistically significant findings, the standard errors in Table 3 warrant caution about the point estimates. For instance, the issue discussed above regarding the point estimate of an eleven years difference in median lifespan between low and high SES athletes for the younger cohorts, comes with a 95% confidence interval of about 3 to 19 years (not reported in the tables).

Fifth, the decrease in the share of high SES athletes with increasing year of birth (45% for the older versus 36% for the younger cohort groups; not reported in the tables) could reflect that athletes who participated in the earlier Olympics, when sport as a leisure activity was a luxury good, were more often from wealthier families. This could have affected the comparability of the high SES category across cohort groups. If wealthier is associated with healthier, this might have caused an underestimation of the steepening of the SES-lifespan gradient.

Sixth, the athletes are not followed from birth but from the time they participated in the Olympics. Especially the older cohorts’ early years could have been in times when there was a significant SES gradient in infant and child mortality [31,32], so that the group that reached adulthood, may already have been a selected one. Hence, while this study’s data shows no significant SES-lifespan gradient for athletes born before 1900, it is possible that the gradient for these cohorts was influenced by differential infant and child mortality. The latter differential mortality has not been taken into account in this paper, and the results are conditional on athletes having reached adulthood and participated in the Olympic Games.

### Policy relevance

This paper expressed the steepening of the SES-mortality gradient in terms of median lifespans rather than period life expectancies. Although the data demands for this approach are high, with expectations that one should observe individuals throughout their lives, the study provides policy makers with a historical perspective on how the SES-lifespan gradient has varied across generations. Concerning pension policy in the Netherlands, for instance, the steepening of the SES-lifespan gradient with an increase in the year of birth suggests an increasing disparity between low and high SES individuals across generations in the number of years of state pension receipt after the statutory state pension age, which erodes the redistributive nature of the state pension system [30,33].

Public health policy can, arguably, be warranted to curb the steepening of the SES-lifespan gradient. In search of the root causes of the SES-mortality gradient, which could provide policy instruments for mitigating it, many arguments have been put forward with mixed empirical support, and often based on inferences of associations rather than causal inferences. Such arguments are, for instance, that SES directly affects health or mortality, that behavioral health risk factors such as smoking and obesity affect the education-health gradient, and that psychological stress plays an important role in the SES-health relationship [1,3,34,35]. Concerning the latter argument, increased competition in society and the psychological stress related to dissatisfactory outcomes of competitions, or job-related stress could play a causal role in the rising SES-health gradient [17,36–38].

Universal health coverage and health expenditures can positively affect lifespans [28,39]. One could, therefore, perhaps argue that health or lifespan disparities based on SES, irrespective of their causes, could be reduced by facilitating individual access to health care when needed [4]. In the Netherlands, the welfare state which provides such access has strengthened during the 20^th^ century, introducing universal health care, as well as disability and long-term care insurance programs. While there is some evidence suggesting that the Dutch welfare state has had a mitigating effect on the SES-health gradient [40], it has not prevented the steepening of the SES-lifespan gradient over time. Future research, therefore, could be directed at determining the importance of behavioral factors in combination with long-term societal changes, also other than those related to the welfare state, for the development of the SES-lifespan gradient [3,41].

### Ethics statement

This study has been approved by the Ethics Committee of the Faculty of Law, Economics and Governance at Utrecht University.

## Supporting information

S1 Fig Lifespan distributions of Olympic athletes and the general population by Olympic Games in which they first participated. (DOCX)

S1 Table Number of Dutch Olympic athletes and percentages female and deceased by Olympic Games in which they first participated. (DOCX)

S2 Table Lifespan distribution by occupation, type of sport and medal position. (DOCX)

S3 Table Statistical tests on the differences in the SES-mortality gradient between cohort groups. (DOCX)

S4 Table Statistical tests on the differences in the SES-lifespan gradient between cohort groups. (DOCX)
