# Supplementary material for: The socio-economic status gradient in median lifespan by birth cohorts: Evidence from Dutch Olympic athletes born between 1852 and 1947
Source: PLoS One. 2019 Dec 11;14(12):e0226269. doi: 10.1371/journal.pone.0226269 (PMC6905560; doi:10.1371/journal.pone.0226269)
Supplement: S1 Fig — (DOCX) [file pone.0226269.s001.docx]

**S1 Fig****. Lifespan distributions of Olympic athletes and the general population^a^ by Olympic Games** **in which they first participated.**

Q25 is the first quartile, Q50 the second quartile (median), and Q75 is the third quartile of the lifespan distribution. Due to low numbers of observations (S1 Table), athletes of the 1900 and 1908 Olympics are included in the 1906 Olympics, athletes of the 1932 Olympics are included in the 1936 Olympics, and athletes of the 1956 Olympics are included in the 1952 Olympics. No lifespan quartiles can be reported for athletes of the 1964 Olympics as most of them were still alive in December 2018.

^a^ Based on a weighted average of annual death rates of the athletes’ national cohorts and gender, conditional on having reached age 26 (the average age at which athletes participated in the Olympics).
