# Supplementary material for: The socio-economic status gradient in median lifespan by birth cohorts: Evidence from Dutch Olympic athletes born between 1852 and 1947
Source: PLoS One. 2019 Dec 11;14(12):e0226269. doi: 10.1371/journal.pone.0226269 (PMC6905560; doi:10.1371/journal.pone.0226269)
Supplement: S1 Table — (DOCX) [file pone.0226269.s002.docx]

**S1 Table Number of Dutch Olympic athletes and percentages female and deceased by Olympic Games in which they first participated**

|  |  | Athletes | Female | Deceased  by 31-12 2018 |
| --- | --- | --- | --- | --- |
| Olympic Games | | Freq. | % | % |
| 1900 | Paris | 42 | 0 | 100 |
| 1906 | Athens | 16 | 0 | 100 |
| 1908 | London | 86 | 0 | 100 |
| 1912 | Stockholm | 26 | 0 | 100 |
| 1920 | Antwerp | 87 | 1 | 100 |
| 1924 | Paris/Chamonix | 111 | 5 | 100 |
| 1928 | Amsterdam/St. Moritz | 132 | 8 | 100 |
| 1932 | Los Angeles/Lake Placid | 11 | 27 | 100 |
| 1936 | Berlin / Garmisch-Partenkirchen | 117 | 12 | 100 |
| 1948 | London/St. Moritz | 85 | 5 | 91 |
| 1952 | Helsinki/Oslo | 53 | 9 | 70 |
| 1956 | Melbourne, Stockholm / Corina d'Ampezzo | 4 | 25 | 75 |
| 1960 | Rome/Squaw Valley | 77 | 6 | 40 |
| 1964 | Tokyo/Innsbruck | 87 | 9 | 20 |
| 1900–1964 | | 934 | 6 | 85 |

About 15% of the athletes participated in more than one Olympics. Relatively few athletes participated in the 1932 Olympics because of financial constraints, and no Dutch athletes participated in the 1956 Summer Olympics because of an official boycott in response to the Soviet Union’s invasion of Hungary.
