# Supplementary material for: The socio-economic status gradient in median lifespan by birth cohorts: Evidence from Dutch Olympic athletes born between 1852 and 1947
Source: PLoS One. 2019 Dec 11;14(12):e0226269. doi: 10.1371/journal.pone.0226269 (PMC6905560; doi:10.1371/journal.pone.0226269)
Supplement: S2 Table — (DOCX) [file pone.0226269.s003.docx]

**S2 Table Lifespan distribution by occupation, type of sport and medal position**

|  | *n* | Lifespan (in years) | |  |
| --- | --- | --- | --- | --- |
|  |  | 25th percentile | 50th percentile | 75th percentile |
| All athletes | 934 | 67 | 78 | 86 |
| *Occupation (SES category)* | |  |  |  |
| Military, e.g. sergeant (Low SES) | 16 | 60 | 69 | 77 |
| Unskilled (Low SES) | 137 | 66 | 76 | 84 |
| Military, e.g. lieutenant (Medium SES) | 31 | 50 | 75 | 85 |
| Craftsman (Medium SES) | 128 | 71 | 81 | 87 |
| Shopkeeper (Medium SES) | 83 | 67 | 77 | 87 |
| Office employee (Medium SES) | 88 | 70 | 82 | 86 |
| Teacher (Medium SES) | 68 | 71 | 82 | 88 |
| Military, e.g. colonel (High SES) | 32 | 62 | 77 | 83 |
| Administrator (High SES) | 44 | 70 | 82 | 88 |
| Merchant (High SES) | 61 | 68 | 78 | 87 |
| Director (High SES) | 74 | 65 | 76 | 85 |
| Engineer (High SES) | 85 | 67 | 81 | 87 |
| Lawyer (High SES) | 29 | 59 | 65 | 83 |
| Doctor (High SES) | 58 | 66 | 76 | 89 |
| *Type of sport* |  |  |  |  |
| Athletics & Gymnastics | 149 | 66 | 79 | 82 |
| Power sports^a)^ | 85 | 66 | 76 | 85 |
| Field hockey & football | 144 | 67 | 76 | 90 |
| Rowing & canoeing | 131 | 69 | 81 | 86 |
| Fencing | 55 | 64 | 77 | 88 |
| Swimming & water polo | 116 | 69 | 80 | 87 |
| Cycling & speed skating | 99 | 72 | 80 | 88 |
| Sailing | 44 | 68 | 79 | 86 |
| Other sports^b)^ | 111 | 68 | 78 | 87 |
| *Medal position* |  |  |  |  |
| No medal | 685 | 68 | 79 | 87 |
| Bronze medalist | 131 | 67 | 76 | 85 |
| Silver medalist | 72 | 66 | 76 | 86 |
| Gold medalist | 46 | 65 | 77 | 84 |
| Participated one time | 772 | 67 | 78 | 86 |
| Participated multiple times | 162 | 67 | 78 | 85 |

*n* = number of athletes. Log-rank tests reject equality of survivor functions by *Occupation* (p-value=0.002) and by *Type of sport* (p-value=0.028) and also rejects equality by *Medal position* (p-value=0.08) and by whether or not *Participated multiple times* (p-value=0.26). ^a)^ Power sports include boxing, tug of war, wrestling, bobsledding, weight lifting, and judo. ^b)^ Other sports include gymnastics, shooting, archery, tennis, volleyball, equestrianism, figure skating, alpine skiing, and art.
