# Supplementary material for: The socio-economic status gradient in median lifespan by birth cohorts: Evidence from Dutch Olympic athletes born between 1852 and 1947
Source: PLoS One. 2019 Dec 11;14(12):e0226269. doi: 10.1371/journal.pone.0226269 (PMC6905560; doi:10.1371/journal.pone.0226269)
Supplement: S3 Table — (DOCX) [file pone.0226269.s004.docx]

**S3 Table** **Statistical tests on the differences in the SES-mortality gradient between cohort groups**

|  | Older birth  cohorts  1852-1899 | Middle birth  cohorts  1900-1919 | Younger birth  cohorts  1920-1947 |
| --- | --- | --- | --- |
|  | Coeff. | Coeff. | Coeff. |
| Covariate: Socio-economic status (SES) | (*SE*) | (*SE*) | (*SE*) |
| Reference category: Medium SES | [p-value] | [p-value] | [p-value] |
| *Panel A. Interactions with each cohort group* | | |  |
| Low SES | -0.21 | 0.44** | 0.57** |
| (interactions with cohorts) | (0.17) | (0.18) | (0.25) |
|  | [0.22] | [0.02] | [0.03] |
| High SES | 0.12 | 0.06 | -0.48** |
| (interactions with cohorts) | (0.14) | (0.14) | (0.23) |
|  | [0.39] | [0.68] | [0.04] |
| H_0_: No associations with SES (p-value) | 0.23 | 0.05** | 0.00*** |
| *Panel B. Reference group: 1852-1899 cohorts* | | |  |
| Low SES | -0.21 | 0.65*** | 0.77** |
| (interactions for the two non-reference groups) | (0.17) | (0.25) | (0.30) |
|  | [0.22] | [0.01] | [0.01] |
| High SES | 0.12 | -0.06 | -0.60** |
| (interactions for the two non-reference groups) | (0.14) | (0.20) | (0.27) |
|  | [0.39] | [0.76] | [0.03] |
| H_0_: No associations with SES (p-value) | 0.23 | 0.02** | 0.00*** |
| *Panel C. Reference group: 1900-1919 cohorts* | | |  |
| Low SES | -0.65*** | 0.44** | 0.12 |
| (interactions for the two non-reference groups) | (0.25) | (0.18) | (0.31) |
|  | [0.01] | [0.02] | [0.69] |
| High SES | 0.06 | 0.06 | -0.54 |
| (interactions for the two non-reference groups) | (0.20) | (0.14) | (0.28) ** |
|  | [0.76] | [0.68] | [0.05] |
| H_0_: No associations with SES (p-value) | 0.02** | 0.10* | 0.05** |

Three mortality rate models with interactions between cohort groups and SES (and all other covariates) are estimated using the full sample of 934 athletes. The first model, Panel A results, includes interactions between the three cohort groups and SES. These results are identical to those in Table 2 that are based on estimations of the SES-mortality gradient by cohort group. The other two models, Panels B and C results, include SES (without an interaction) and interaction terms for SES of the two non-reference cohort groups. In panels B and C test results are presented concerning if the associations between SES and mortality differ between two cohort groups (the null-hypotheses are each denoted by H_0_). Levels of significance: *** p<0.01 ** p<0.05, * p<0.1.
