# Supplementary material for: The socio-economic status gradient in median lifespan by birth cohorts: Evidence from Dutch Olympic athletes born between 1852 and 1947
Source: PLoS One. 2019 Dec 11;14(12):e0226269. doi: 10.1371/journal.pone.0226269 (PMC6905560; doi:10.1371/journal.pone.0226269)
Supplement: S4 Table — (DOCX) [file pone.0226269.s005.docx]

**S4 Table** **Statistical tests on the differences in the SES-lifespan gradient between cohort groups**

| Cells: all predictions are in years. | Older birth  cohorts  1852-1899  (Reference group) | Middle birth  cohorts  1900-1919 | Younger birth  cohorts  1920-1947 |
| --- | --- | --- | --- |
|  | Prediction | Prediction | Prediction |
| Difference relative to the older cohorts in the median lifespan of the general population | 0.00 | 1.00 | 5.00 |
| Difference relative to the older cohorts in the median lifespan of medium SES athletes | 0.00 | 5.15 | 11.70 |
| *Differences between cohort groups of the* | Prediction | Prediction | Prediction |
| *predictions in Table 3 of medium lifespan* | (SE) | (SE) | (SE) |
| *differences between SES categories.* | [p-value] | [p-value] | [p-value] |
| Median lifespan of medium SES | 0.00 | 4.15 | 6.70* |
| athletes minus that of the general | ( - ) | (3.05) | (3.88) |
| population | [ - ] | [0.174] | [0.084] |
| Median lifespans of low SES athletes | 0.00 | -7.83*** | -8.68** |
| minus that of medium SES athletes | ( - ) | (3.01) | (3.61) |
|  | [ - ] | [0.009] | [0.016] |
| Median lifespan of high SES athletes | 0.00 | 0.40 | 6.06** |
| minus that of medium SES athletes | ( - ) | (2.37) | (2.86) |
|  | [ - ] | [0.866] | [0.034] |

Presented are the differences between cohort groups of the predictions in the different columns of Table 3 with the first column as a reference (the older birth cohorts). These differences in predictions across cohort groups are based on the estimation results of Eq. (1) with interactions between cohort groups and SES (and all other covariates) using the full sample of 934 athletes (S3 Table). Standard errors in parentheses and p-values in brackets are provided. Median lifespan predictions are conditional on athletes having reached age 26 (i.e. the average age at which athletes participated in the Olympics). Levels of significance: *** p<0.01 ** p<0.05, * p<0.1.
